# The Primo Vascular Structures Alongside Nervous System: Its Discovery and Functional Limitation

**DOI:** 10.1155/2013/538350

**Published:** 2013-03-31

**Authors:** Eun-sung Park, Hee Young Kim, Dong-ho Youn

**Affiliations:** ^1^Department of Oral Physiology, School of Dentistry, Kyungpook National University, Daegu 700-412, Republic of Korea; ^2^Department of Quality Control, Koatech, Pyeongtaek, Gyeonggi-do 451-864, Republic of Korea; ^3^Department of Physiology, College of Korean Medicine, Daegu Haany University, Daegu 706-060, Republic of Korea

## Abstract

The primo vascular structures comprising primo nodes and vessels (originally called Bonghan corpuscles and ducts, resp.) have recently been suggested to be the anatomical correlate of acupuncture, a therapeutic technique used in oriental medicine. Although the primo vascular structures have been observed in many parts of animals, including the nervous system, using anatomical methodologies, its physiological functions are still unclear. This paper summarizes the reports on the primo vascular structures, particularly in the nervous system and its surroundings, as well as the electrophysiological properties of cells in the primo nodes. In addition, recent reports examining the potential roles of the primo vascular structures in acupuncture are discussed. This review raises some fundamental questions and, at the same time, highlights the potential physiological roles of the primo vascular structures in acupuncture.

## 1. Introduction

Acupuncture is a centuries-old therapeutic technique in oriental medicine, but its therapeutic mechanism is unclear. Currently, acupuncture is becoming popular in Western countries, and NIH (National Institutes of Health, USA) has also agreed that acupuncture is effective or at least useful in the treatment of 13 conditions, including low back pain, fibromyalgia, and stroke rehabilitation [1]. During acupuncture, fine needles are inserted through the skin and muscle at certain acupuncture points (so-called “acupoints”) on the body of the patient, which are located mainly along the “meridians” [2]. In addition to the different principles used in oriental medicine, many studies using modern scientific (Western style) methodologies have attempted to define the anatomical structures of the acupoints and meridians that make the unique oriental technique work both physiologically and clinically in the bodies of humans or animals [2]. Soh's group has examined the primo vascular structures, which was originally coined by Kim Bonghan in 1963, as potential anatomical structures for the acupoints and meridians. By appreciating the various conventional staining or advanced microscopic imaging methods (Table 1), the existence of the primo vascular structures was reported in various areas of the body, including the nervous system [3]. The primo vascular structures, which consist of primo nodes (previously called Bonghan corpuscles) and primo vessel (previously called Bonghan duct) [3], were categorized according to its location as follows: superficial (in the skin), intravascular, extravascular, organ-surface, intraorgan, and neural primo vascular systems. The neural primo vascular system is designated when it exists inside or adjacent to the brain and spinal cord and is accompanied by the outer surface of peripheral nerves [3].

This paper summarizes the discovery of the primo vascular structures alongside central and peripheral nervous system and discusses the technical concerns encountered in staining the primo vascular structures and its potential functional roles in acupuncture.

## 2. Discovery of the Primo Vascular Structures Alongside Central and Peripheral Nervous System

Although the primo vascular structures have been found in various areas of the body, such as the blood vessels, lymphatic vessels, and enteric organs, its identification in the area of the peripheral and central nervous system using various staining methods was only recent (Table 1). In the rat brain and spinal cord, the injection of fluorescent nanoparticles into the lateral ventricle stained the primo vessels floating in the cerebrospinal fluid of the 4th ventricle, as well as those lying alongside the blood vessels in the subarachnoid space in the dorsal surface of the spinal cord [4]. Similarly, the primo nodes and primo vessels on the arachnoid mater and primo node in the 4th ventricle of the rat brain were stained with trypan blue [5]. In the rabbit nervous system, primo vessels were observed in the mesencephalic aqueduct and 4th ventricle of the brain as well as in the central canal of the spinal cord by confocal laser scanning microscopy and electron microscopy [6]. Their structures contained DNA particles, other microparticles, and rod-shaped nuclei encircled by helix-shaped actins. In addition, primo vessels were observed in the 3rd and 4th ventricles and the central canal of the rabbits using hematoxylin staining, and the rod-shaped nuclei structure of the primo vessels was visualized by nucleus-specific staining dyes, such as 4′,6′-diamidino-2-phenylindole, propidium iodide, and yoyo-1 [7]. Interestingly, the primo vessel was also observed for the first time in the subarachnoid space of the thoracic spinal nerve area in a pig [11]. Overall, these studies suggest the existence of the primo vascular structures in the brain and spinal cord, particularly in the ventricles, central canal, and arachnoid spaces (Table 1). On the other hand, the primo vessels were observed in the peripheral nervous system of rats using trypan blue staining [5] or fluorescent nanoparticles [12], mostly in the epineurium, perineurium, and endoneurium of the sciatic nerve.

## 3. Technical Considerations in Staining Methods

A range of staining dyes or fluorescent nanoparticles have been used to identify the structure of the primo vascular system in the nervous system and its surroundings (Table 1). On the other hand, the mechanism of staining with these dyes is based frequently on nonspecific binding because most dyes use the electrostatic interactions between the charges of the dyes and the charges of tissues. For example, hematoxylin, alcian blue and trypan blue used to stain the primo vascular structures forms salt linkages with the acidic or basic groups of various organic molecules in intracellular or extracelluar structures.

Hematoxylin is one of the most common dyes used for nuclear staining in histology. The application of hematoxylin to the 4th ventricle of frozen brain revealed the primo vessel that appeared like pale blue-stained thread along the midline of the fourth ventricle [6]. The primo vessel contained many fragmented DNA particles and rod-shaped nuclei (10~20 *μ*m in size) with irregular distribution; the DNA particles or nuclei were stained dark blue. Thus, the primo vessel appeared as a thin thread with tiny blue dots under high power magnification. Even though the use of hematoxylin revealed the structure of the primo vessel, it should be noted that its value is not limited to the nuclear staining because it can be used to detect intracellular substances (e.g., chromosomes, keratohyalin), extracellular substances (e.g., elastin), ground substances (e.g., cement lines in bone), minerals (e.g., calcium, copper, etc.), and the central nervous system (e.g., myelin, neuroglia fibers, etc.) [13].

S. Lee and C. Lee [14] reported that alcian blue could visualize primo vessels and nodes above the pia mater of the brain without staining surrounding blood vessels. Because alcian blue is a water soluble copper phthalocyanine that easily binds to acidic groups, such as acid mucosubstances and acetic mucins normally found in blood vessel walls [13], a careful use of alcian blue is recommended, especially in central nervous system with microvascular networks. Trypan blue is used to selectively stain dead cells or tissues because the chromophore is negatively charged and does not penetrate the cell membrane unless the membrane is damaged. If the dye enters live cells, it is exocytosed actively. Accordingly, live cells or tissues with intact cell membrane are not stained by trypan blue. Therefore, staining of the primo structures with this dye [3] might be due to long-term exposure of the dye because its increased negative charge under basic conditions can bind to the fibril in the cytosol after membrane penetration or inclusion of dead/dying cells in primo vessels. DiI (Molecular probes, OR, USA) is a fluorescent lipophilic carbocyanine dye bound to the membrane phospholipids that is used typically as a retrograde membrane tracer by neuroscience researchers. Therefore, DiI can bind to any cellular components containing phospholipids. Another technique to stain the primo structure is to use fluorescent nanoparticles composed of cobalt-ferrite embedded in silica shells with rhodamine B on a nanometer scale [4]. On the other hand, the mechanism by which nanoparticles stain specifically the primo vascular structures is unclear. Therefore, the development of staining methods specific to the primo vascular structures is needed to reveal the system as a novel structure supporting the acupuncture mechanism.

## 4. Potential Functions of the Primo Vascular Structures in Central and Peripheral Nervous System

The gross anatomical and histological findings of the primo vascular structures in various areas of the body, including the nervous system, support the anecdotal observations reported by Bonghan Kim, who proposed the primo structures, initially called Bonghan system, as a theory explaining the “meridians” [3]. In ancient oriental medicine, the meridians were believed to be a channel that allows invisible vital energy in live bodies, so-called “Qi,” to flow through [2]. Therefore, he attempted to correlate the primo vascular system to the meridians. Although there is no direct evidence of a relationship between the primo structures and the theory of meridians, or any other functional system, the physiological roles of the primo vascular structures in the nervous system and its surroundings should be examined. For example, Kim reported that the primo vascular structures located in various body parts including the nervous system might supply nutrients to the nervous tissues to be maintained under healthy conditions or be regenerated after damage [3, 15]. This idea is in some way supported by a recent finding that F-actin of the primo vessels in the rat sciatic nerve might allow the structure to contract and to flow the fluid within the primo vessels [5]. On the other hand, the most attractive hypothesis would be that the primo vascular system might play a key role in acupuncture treatment because the primo vascular structures are believed to be the physical presence of acupuncture meridians [3, 7]. Therefore, this section discusses the primo vascular system in terms of its electrophysiological properties and potential relevance with the acupuncture points and meridian. 

The primo nodes, which are found on the surfaces of the internal organs of rats, contain some cells, including macrophages, mast cells, and eosinophils [8]. When measured using whole-cell patch clamp recordings, the cells found in the slices of primo nodes showed large variability in the cell populations, which was characterized by a lower resting membrane potential (−50 ~−10 mV), higher input resistance (200~1600 MΩ), and lower capacitance (5~20 pF) compared to the neurons in the hypothalamic paraventricular nucleus [9, 10]. In addition, no active components in the cell membrane properties were observed in the preparation of primo nodes [9, 10], suggesting that the cells found in the live slices of primo nodes are not excitable electrophysiologically [10]. When cells in the primo nodes are classified based on the current-voltage relations and the kinetics of the activation and inactivation of currents generated by the voltage pulses (600 ms) in voltage-clamp mode, one type of cell was found to be sensitive to relatively high concentrations of tetraethylammonium (IC_50_ = ~4.3 mM) [10], indicating that unidentified K^+^ channels may be expressed in these cells. Although no electrophysiological study has attempted to analyze the cells found in the primo nodes of the surroundings of the central or peripheral nervous system, studies using whole-cell patch clamp recordings suggest that the primo nodes do not support the neural mechanism for acupuncture because skin stimulation with fine acupuncture needles might not generate any electrical signals in the primo nodes [2].

The meridian is considered a key concept in oriental medicine including acupuncture, even though its anatomical structures have not been identified. Soh's group proposed that the primo vascular system corresponds to the acupuncture meridian system and examined the correlations between the primo vascular system and acupuncture meridian system. In their previous reports, after injecting a fluorescent dye into the acupoint ST36, the fluorescent network of the primo vascular structures appeared on the layer of the superficial fascia around the stomach meridian from the knee to the tibia [12]. Thin thread-like structures were also observed in hypodermal layer of a rat after staining with trypan blue [16]. Unfortunately, there was no evidence of the role of primo vascular structures in the effect of acupuncture. Recently, Wang et al. (2012) [17] examined the effects of stimulation of the primo vessels on gastric motility as well as the roles of the primo vessels in mediating the effects of acupuncture on gastric motility [18, 19]. In their results, direct electrical stimulation of the primo vascular structures on the stomach did not affect the gastric motility, and the inhibitory or stimulatory effects induced by acupuncture at CV12 or ST36 were unchanged after the primo vascular structures had been cut. They suggested that the primo vascular structures are not involved in the acupuncture modulation of gastric motility. The possibility that the primo vascular system might utilize other biological processes to achieve the therapeutic effects of acupuncture on gastric disorders cannot be excluded because Wang et al.'s study used only one measurement of gastric motility [17] and the primo vascular system was implicated in a range of biological processes, such as the immune responses, hormone, and regeneration [20]. Therefore, the functional roles of the primo vascular system in the effects of acupuncture on gastric motility are unclear.

Although Soh's group has reported that the primo vascular system mediates the transmission of acupuncture signals as an acupuncture meridian, many studies have shown that the afferent signals of acupuncture are conveyed via the peripheral nerve rather than the “Qi,” meridian or other routes. The effects of acupuncture are blocked by a pretreatment with local anesthetic drugs around the acupoints [21, 22]. The responses elicited by stimulating the acupoints are blocked when the nerve lying under the acupoint is cut [23, 24], and direct stimulation of the nerves innervating acupoints also has a similar effect to that of acupuncture [24, 25]. Apart from lack of attempt to block selectively either nerves or primo vessels, it might be spoken up that further studies on the functional relationship between the primo vascular system and meridian are required for the suggestion that the primo vascular system, including primo vessel and nodes, corresponds to acupuncture meridian system and thus mediate the acupuncture effects. Moreover, slow flow rate (0.3 mm/sec) through the primo vessel [26], which in turn may take more than 400 sec to travels through a 12 cm long primo vessel in internal organs, should be reconciled with the fact that the time taken to initiate the response after stimulating the acupoints is immediate or occurs in several minutes [27, 28].

## 5. Conclusion

The primo vascular system has been proposed as the anatomical structure that mediates the effects of acupuncture. Nevertheless, the lack of specific staining methods that can distinguish the structures from other tissues has thwarted the easy and reproducible detection of the entire connections in the body, building an obstacle for a reliable and functional analysis of the system. Thus, an immunohistochemical staining based on molecular markers specific to the primo vascular structures needs to be developed in the future, giving support to them for the normal anatomical structures, not like pathological products. In addition, the finding that the cells in the primo nodes have only passive membrane properties, suggests that the primo vascular system might be unsatisfactory for mediating the rapid effects of acupuncture. Therefore, functional studies, such as cutting the primo vascular system selectively [22], might help understand the roles of the system in acupuncture. Moreover, identifying the molecular contents in the primo vascular structures as well as their functional release to needling in acupuncture will put the primo vascular system in the position of the real anatomical structure of acupuncture.

## Figures and Tables

**Table 1:** Primo nodes and vessels in the surroundings of the peripheral and central nervous system.

Areas	Species and sites	Dyes
3rd or 4th ventricles	Rat [3, 4]; rabbit [5, 6]	Fluorescent nanoparticles, hematoxylin, Acridine orange, propidium iodide, DiI, Perchlorate, DAPI, and yoyo1
Central canal of the spinal cord	Rabbit [5, 6]	Hematoxylin, Acridine orange, propidium iodide, DiI, Perchlorate, DAPI, and yoyo1
Subarachnoid space	Rat [3] and pig [7] spinal cords; rat brain [4]	Fluorescent nanoparticles, DAPI, and Phalloidin
Venous sinus	Rat cerebrum [8]	Chromium hematoxylin and DAPI
Surroundings of the brain	Rat cerebrum [9], pineal body [9], and spinal cord [4, 9]; rat [4, 9] and rabbit [10] cerebellums	Alcian blue, DAPI, trypan blue, Phalloidin, and DAPI
Surroundings of the peripheral nerve	Epineurium, perineurium, or endoneurium in rat sciatic nerve [4, 11]	Fluorescent nanoparticles and DAPI

Technical notes for dyes: DAPI, for nuclei; Phalloidin, for nuclei and F-actin; Acridine orange, for DNA particles; Propidium iodide, for nucleus and F-actin; DiI, for membrane phospholipid; Perchlorate, for microparticles inside the nerve primo vessel; Fluorescent nanoparticles, cobalt-ferrite embedded in silica shells with rhodamine B as a contrast agent during MRI.
